# Geographic Variations in the Risk of Emergency First Dialysis for Patients with End Stage Renal Disease in the Bretagne Region, France

**DOI:** 10.3390/ijerph16010018

**Published:** 2018-12-21

**Authors:** Cindy M. Padilla, Maxime Raffray, Adélaïde Pladys, Cécile Vigneau, Sahar Bayat

**Affiliations:** 1 Univ Rennes, EHESP, REPERES (Recherche en pharmaco-épidémiologie et recours aux soins) – EA 7449, F-35000 Rennes, France; maxime.raffray@ehesp.fr (M.R.); adelaide.pladys@ehesp.fr (A.P.); sahar.bayat-makoei@ehesp.fr (S.B.); 2 CHU Pontchaillou, Service de Néphrologie, 35033 Rennes, France; cecile.vigneau@chu-rennes.fr; 3 Univ Rennes, EHESP, Inserm, Irset (Institut de recherche en santé, environnement et travail) – UMR_S 1085, F-35000 Rennes, France

**Keywords:** end stage renal disease, emergency first dialysis, spatial analysis, socio-demographic, patient and municipality level

## Abstract

Emergency first dialysis start considerably increases the risk of morbidity and mortality. Our objective was to identify the geographic variations of emergency first dialysis risk in patients with end-stage renal disease in the Bretagne region, France. The spatial scan statistic approach was used to determine the clusters of municipalities with significantly higher or lower risk of emergency first dialysis. Patient data extracted from the REIN registry (sociodemographic, clinical, and biological characteristics) and indicators constructed at the municipality level, were compared between clusters. This analysis identified a cluster of municipalities in western Bretagne with a significantly higher risk (RR = 1.80, *p* = 0.044) and one cluster in the eastern part of the region with a significantly lower risk (RR = 0.59, *p* < 0.01) of emergency first dialysis. The degree of urbanization (the proportion of rural municipalities: 76% versus 66%, *p* < 0.001) and socio-demographic characteristics (the unemployment rate: 11% versus 8%, *p* < 0.001, the percentage of managers in the labor force was lower: 9% versus 13% *p* < 0.001) of the municipalities located in the higher-risk cluster compared with the lower-risk cluster. Our analysis indicates that the patients’ clinical status cannot explain the geographic variations of emergency first dialysis incidence in Bretagne. Conversely, where patients live seems to play an important role.

## 1. Introduction

In 2011, the incidence rates of end stage renal disease (ESRD) varied considerably between European countries, from 85 people per million (ppm) in Finland to 226 ppm in Portugal [1,2]. However, the highest rates were reported in the USA and Mexico with 362 and 527 ppm, respectively [3]. In mainland France, the rate increased from 144 to 159 ppm between 2005 and 2014 [4].

ESRD management is a major public health concern. In 2016 in France, 32% of incident hemodialyzed patients with ESRD started dialysis in emergency. This has major negative effects on both outcomes and costs [5,6]. Indeed, emergency first dialysis considerably increases the risk of morbidity and mortality [7,8], reduces the patient quality of life [7,9,10,11,12], and increases the hospital stay length [9,13]. A previous study in France analyzed the characteristics of patients with ESRD who started dialysis in emergency conditions, and found that their one-year survival and placement on the kidney transplant waiting list rates were lower than those of patients who received planned first dialysis (survival rate: 74% versus 87%, *p* < 0.0001; placement on the waiting list rate: 22% versus 31%, *p* < 0.0001) [14].

Moreover, good coordination between general practitioners (GPs) and nephrologists during the pre-dialysis period is crucial to minimize the need to start dialysis as an emergency [7,9,10,11,12]. The leading cause of emergency dialysis start is late patient’s referral to a nephrologist. For instance, silent chronic kidney disease (CKD) could be undiagnosed by the GP and could be discovered only at the end stage. On the other hand, early referral of patients with CKD does not guarantee that dialysis will be initiated in optimal conditions [15,16,17]. Some patients followed by a nephrologist may start dialysis as an emergency because of acute decompensation. Indeed, a combination of patient factors (being older, belonging to a minority group, being less educated, being uninsured, suffering from multiple comorbidities) and health system factors has been associated with late referral [18] and could be linked to emergency dialysis start. Healthcare access limitations, such as distance between home and health professionals (GP or nephrologist), difficulty in obtaining or paying for medical care, type of healthcare provider, and patient–provider interactions [19,20,21,22], might influence the patient’s care trajectory [19].

This study analyzed the geographic variations of the risk of emergency first dialysis for patients with ESRD in the Bretagne region, France. The objectives were: (i) to identify the clusters of municipalities with significantly higher or lower risk of emergency first dialysis, and (ii) to compare the patient level and municipality level characteristics in these clusters of municipalities.

## 2. Materials and Methods

### 2.1. Study Setting

**Figure 1:**
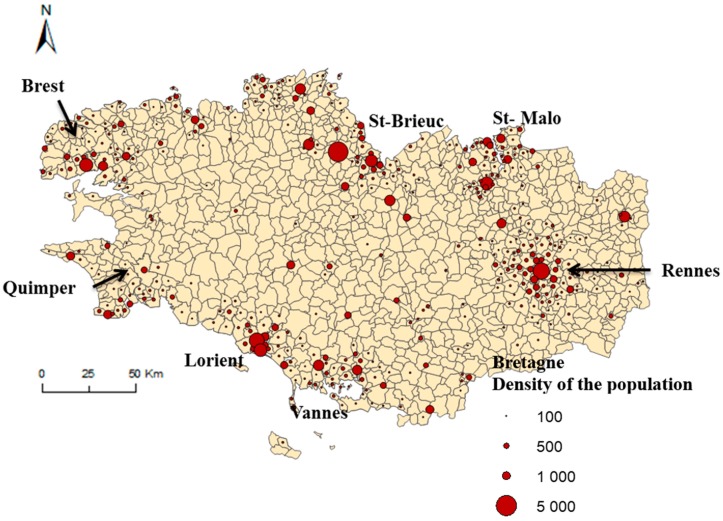
Major cities in Bretagne. Geographic representation of the density of the population by municipalities.

The study was carried out in the Bretagne administrative region (3,276,543 inhabitants in 2014) that is located in western France, and is subdivided in 1270 municipalities. The Bretagne region is an attractive region and all the departments participating in this attractiveness. As everywhere in France, young people leave the region for their studies and population having a job or looking for a job come to live in the region. The distribution of the population is homogeneous, nevertheless the urban cities (Figure 1) attract the young people and the coastline (mainly in Morbihan department) attracts young retirees. Bretagne region is particular relevant for studying access to healthcare because it contains both rural and urban areas (see major urban cities in Figure 1) [23]. The degree of rurality is a proxy of healthcare access and a major risk factor of an increase rate of incidence of ESRD. Moreover, the incidence rate in Bretagne of ESRD patients who start dialysis in emergency is close to the national incidence rate in France (25% vs. 34% at the national level).

### 2.2. Study Population

All adult patients (i.e., older than 17 years of age) who lived in Bretagne and started dialysis in Bretagne between 1 January 2009 and 31 December 2015 were extracted from the French national registry ‘Réseau Epidémiologique et Information en Néphrologie’ (REIN) [6]. The French REIN registry collects data on all patients with ESRD undergoing RRT (dialysis or kidney transplantation) and living in mainland France or in its overseas districts. It does not include patients with a diagnosis of acute renal failure (i.e., patients who recover all or some renal function within 45 days, or are considered by experts to have had acute kidney failure for less than 45 days before death). The registry began in 2002 and progressively expanded over the entire French territory: mainland regions first, then overseas territories to include the entire country since 2011. The registry covers 100% of patients with ESRD in each participating region. Its organizational principles and quality control have been described elsewhere [6]. Each year, the regions for which complete data are available are analyzed to estimate the national level of each indicator. The criteria used to define an unplanned dialysis initiation also differ across studies [13] Fortunately, there is a consensus definition in France of referral that qualifies a patient as “unplanned or emergency” onto dialysis. In the REIN registry, the label “emergency first dialysis start” describes the first dialysis session performed immediately (within 24 h) after the detection of a life-threatening risk by a nephrologist. Among 2740 patients who started dialysis in Bretagne between 1 January 2009 and 31 December 2015, 101 patients (3.5%) had no information on the type of first dialysis (emergency vs. planned), 1974 patients had planned first dialysis start and 665 had emergency first dialysis start.

Subjects involved in our study were extracted from the French REIN registry which received agreement from the CNIL (Commission Nationale de l’Information et des Libertés) in 2010 (agreement number: 903188 Version 3). Verbal informed consent to participate was obtained from all subjects involved. This study approved by the French Biomedecine Agency included patients’ information which has been anonymized and de-identified directly in the database and before the extraction for analysis.

### 2.3. Data Collection

Data were collected at two levels: patient- and municipality-level. The distributions of the patient-level data were presented in Table 1 and Table 2 according to their municipality of residence group. The distributions of the municipality-level data were presented in Table 3 and Table 4.

### 2.4. Patient-Level Factors

Patient-level data were extracted from the REIN registry.

The clinical and biological data at dialysis start included: primary renal disease classified in six categories (glomerulonephritis, pyelonephritis, diabetic nephropathy, hypertensive and vascular nephropathy, polycystic kidney disease, and others (means unknown and unclassifiable nephropathy)); comorbidities, including respiratory disease, active malignancy, hepatic disease, diabetes, cardiovascular diseases (coronary artery disease, peripheral vascular disease, congestive heart failure, arrhythmia, aortic aneurism, and cerebrovascular disease), physical disabilities (physical impairment of ambulation, para- or hemi- plegia, blindness, member amputations), and psychiatric disorders; albumin (<30, ≥30 g/dL) and hemoglobin (<10, 10–12, >12 g/dL) rates; body mass index (underweight and normal: BMI < 25, overweight: BMI ≥ 25, obese: BMI>30); and smoking status (non-smoker, current/former smoker).

The socio-demographic data included sex, age (in three groups: <45, 45–75, and older than 75 years of age), patient’s activity at dialysis start (inactive: student, retired, and homemaker; active: unemployed, full-time, and part-time employed), and municipality of residence.

The data related to the patient’s follow-up at first dialysis were: date of first dialysis, temporary access with a central venous catheter versus permanent access with fistula, number of nephrologist consultations in the year prior to dialysis initiation (less than three consultations versus at least three consultations), and time spent in transport to go to the dialysis facility and the type of transport (ambulance, light sanitary vehicle, taxi, car, other).

### 2.5. Municipality-Level Factors

#### 2.5.1. Socio-Demographic Variables

National Institute of Statistics and Economic Studies (INSEE) data from the national census of 2014 were used to construct an array of social, economic and demographic indicators at the municipality level. These variables can be divided in seven domains: occupation, education, unemployment, immigration status, housing, residential mobility, and resources.

#### 2.5.2. Degree of Urbanization

The degree of urbanization at the municipality level was calculated using an approach inspired from the study by Van Eupan et al. [24]. Three classes were established: rural, peri-urban, and urban. This indicator was calculated in three consecutive steps: (1) the classification of each municipality based on the Organization for Economic Co-operation and Development (OECD) typology [25], using population density criteria; (2) the land cover typology [26], using natural and artificial area criteria; and (3) the combination of the population density and land cover criteria to obtain the final rural/urban classification.

#### 2.5.3. Potential Healthcare Offer

Two indicators were constructed: (1) GPs’ density (i.e., number of GPs per 10,000 inhabitants) in each municipality; (2) proportion of municipalities with at least one dialysis facility within the municipality, using INSEE data from the 2014 national census [27].

### 2.6. Analysis

#### 2.6.1. Spatial Analysis

The geographic variations of the risk of emergency first dialysis for patients with ERSD was evaluated using a spatial scan statistic method [28,29]. This is a cluster detection test that can identify the location of clusters and evaluate their statistical significance. The spatial analysis was done using the SaTScan software, version 9.4 [28].

In this approach, the null hypothesis (H0) tested was that the risk of starting dialysis in emergency (adjusted by age and sex) occurred randomly throughout the Bretagne region. In other words, the relative risk (ratio of the observed-to-expected sex- and age-adjusted patients) was constant over the whole area. The alternative hypothesis (H1) was that the adjusted risk of dialysis in emergency was higher within a given municipality cluster compared with that of municipalities outside the cluster. The number of emergency first dialysis starts in each municipality was assumed to be Poisson distributed. By applying a large number of circles that varied by location and size to contain 1–50% of all population at risk, the spatial scan statistic method counted patients within and outside each circle.

The identification of the clusters is based on a likelihood ratio test, with associated *p*-value obtained using Monte Carlo replications [28]. Monte Carlo approach was used to identify locations with a significantly higher or lower number of events than expected. For hypothesis testing, the SaTScan program generated 999 random replications of the data set under the null hypothesis. The test statistic was calculated for each random replication as well as for the real data set. When the latter was among the 5% highest, the test was significant at the 0.05 level [28]. ArcGis version 9.0 was the geographic information system used for this analysis to view, analyze, and relate data from a spatial (geographic) perspective.

#### 2.6.2. Statistical Analysis

The characteristics of patients living in municipalities with higher and lower risk of emergency first dialysis were compared using Chi-squared test, Exact test and Student’s *t*-test, or Wilcoxon rank test after checking the assumption according to the test. The characteristics of municipalities located inside the higher and lower risk clusters were also compared. All analyses were performed using STATA, release 13 (College Station, TX, USA: StataCorp LP).

## 3. Results

Between 2009 and 2015, 2639 patients started dialysis in Bretagne. Among them, 665 patients (25.2%) had an emergency dialysis start; their mean age was 68 ± 15 years and 68.3% were men (*n* = 454). The incidence of emergency first dialysis was 270 per 100,000 inhabitants.

### 3.1. Geographic Variations

**Figure 2:**
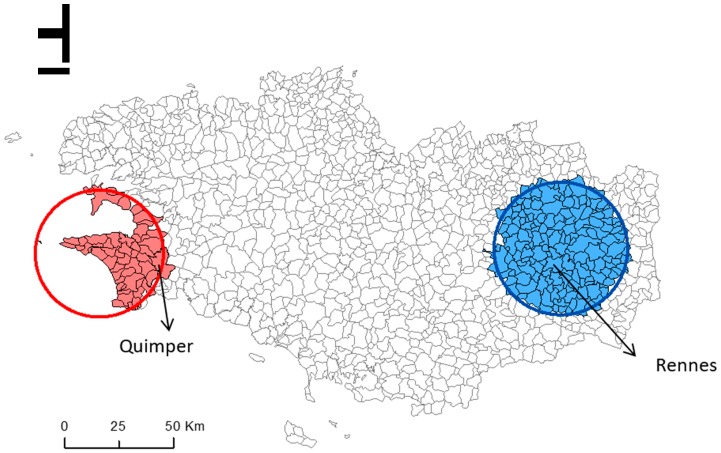
Geographic variations in the risk of emergency first dialysis for patients with end stage renal disease in the 1270 municipalities of the Bretagne region, France. Red circle: cluster of municipalities at higher risk of emergency first dialysis; blue circle: cluster of municipalities at lower risk of emergency first dialysis.

Spatial scan statistic identified one cluster of municipalities with a 1.80 times higher risk of emergency first dialysis (RR = 1.80; *p* = 0.044) compared with all the other Bretagne municipalities, after adjustment for age and sex. This cluster (53 municipalities with 70 patients who started dialysis in emergency) is located in western Bretagne (Figure 2, solid red circle), in the Finistère department (Quimper is the main city).

A second cluster of municipalities showed a significantly lower risk of emergency first dialysis compared with all the other municipalities (RR = 0.59; *p* < 0.01). This cluster (169 municipalities with 78 patients who started dialysis in emergency) is located in eastern Bretagne (Figure 2, solid blue circle), in the Ille-et-Vilaine department (main cities: Rennes and Combourg). The incidence of emergency first dialysis was 434 per 100,000 inhabitants and 133 per 100,000 inhabitants in the higher-risk cluster and in the lower-risk cluster, respectively (Table 1).

### 3.2. Comparison of the Higher- and Lower-Risk Clusters

#### 3.2.1. Patient-Level Factors

Comparison of the patient-level factors in the whole Bretagne region and within the two municipality clusters with significantly higher and lower risk of emergency first dialysis (Table 1 and Table 2) showed that the patients’ clinical (primary renal disease, comorbidities and psychiatric disorders, BMI, smoking status, albumin rate, and hemoglobin rate) and socio-demographic characteristics (age, sex, and activity) were comparable in the two clusters. However, the proportion of physical disabilities was higher among patients living in the lower-risk than in the higher-risk cluster (14.1% versus 2.9% *p* = 0.019).

No difference was observed concerning the vascular access procedure (more than 98% of patients in both clusters had a temporary central venous access for the first dialysis) and also the number of nephrology consultations in the year before dialysis initiation (64.5% of patients in the higher-risk cluster and 73.5% of patients in the lower-risk cluster had more than three consultations; *p* = 0.27).

Conversely, patients living in municipalities in the higher-risk cluster spent longer time in transport on the day of the emergency first dialysis (21 versus 15 min, p = 0.01), and many of them used a light sanitary vehicles (72% vs. 37% in the lower-risk cluster). Conversely, 33% of patients in the lower-risk cluster (versus 12% in the higher-risk cluster; *p* = 0.005) used a taxi, and 10% a car (compared with 0% in the higher-risk cluster; *p* = 0.01).

#### 3.2.2. Municipality-Level Factors

Comparison of the municipality-level variables in the whole Bretagne region and in the two clusters (Table 3 and Table 4) showed that the proportion of stable population (61% of the inhabitants had the same residential address for more than 10 years versus 51.2%, *p* < 0.001) and rural municipalities (76% versus 66%, *p* < 0.001) was higher in municipalities within the higher-risk cluster compared with those in the in lower-risk cluster. The distribution of socio-demographic characteristics highlighted social inequalities between clusters. Indeed, the proportion of managers in the labor force (9% versus 13% *p* < 0.001), of households with at least one car (91% versus 94%, *p* = 0.01), and of people aged 15 years or older who did not go beyond elementary education (28% versus 26%, *p* = 0.006) was higher in municipalities located in the higher-risk than in the lower-risk cluster. Moreover, unemployment was higher (11% versus 8%, *p* < 0.001), and the median income was lower in the higher-risk than in the lower-risk cluster. The GPs’ density and proportion of municipalities with at least one dialysis facility was not significantly different between clusters.

## 4. Discussion

This study analyzed geographic variations in the proportion of patients with ESRD who started dialysis in emergency in the Bretagne region, and identified patient- and municipality-level factors that may contribute to these variations. We identified one cluster of municipalities with higher risk of emergency first dialysis located in western Bretagne, and one cluster with lower risk in eastern Bretagne. Comparing characteristics of the patients living in the high-risk cluster to the patients living in the low risk cluster, we found that this difference was not explained by the patients’ characteristics. Indeed, sociodemographic and clinical features were comparable between clusters. Conversely, the degree of urbanization and factors related to social inequalities were different in the two clusters, and could influence the unequal distribution of patients undergoing emergency first dialysis. Moreover, factors related to the patients’ access to healthcare facilities might also play a role.

The nation-wide geographic variability of ESRD incidence has been described previously in USA [30], Australia [31], and Japan [32]. One of our previous studies showed a cluster with significantly higher risk of ESRD incidence in the western part of Bretagne after adjustment for sex and age [33]. However, the geographic variations of emergency first dialysis and the reasons of these variations were not studied. Here, we identified an area in western Bretagne that includes the city of Quimper with a significantly higher risk of emergency first dialysis, and an area in eastern Bretagne (including the cities of Rennes and Combourg) with a significantly lower risk of emergency first dialysis start during the 2009–2015 period. Previous studies in France investigated the spatial implication of neighborhood characteristics on geographical variations of ESRD incidence [33]; however, to our knowledge, no other study worldwide identified geographic variations of emergency first dialysis incidence and compared the characteristics of patients and municipalities between clusters. For this reason, it is difficult to compare our findings with those of other studies.

Our study revealed no difference in terms of primary renal disease, presence of comorbidities and cardiovascular diseases between patients who started dialysis in emergency and lived in the two clusters. Even if differences are observed in the percentage of patients with no nephrology consultations in the year before dialysis initiation between the two clusters of municipalities, this difference is not significant. Nevertheless, the percentage of patients with physical disabilities was higher in municipalities in the lower-risk cluster. We recommend that patients with physical disabilities have more regular follow-up with specialists and also they are more likely to start with a planned dialysis. In the same way, we found that the percentage of primary renal disease as “Others” (means unknown and unclassifiable nephropathy) was higher in municipalities in the lower-risk cluster.

Conversely, our results show that where one lives might play an important role in the risk of starting dialysis in emergency. First, municipalities located in the higher-risk cluster were more often rural than in the lower-risk cluster as results presented by previous studies [31,33]. A previous study in South Carolina, by Fan et al. demonstrated regional differences of mean incidence rates of ESRD among rural and urban counties [30]. They concluded that lack of access to medical care as lower physician density may explain this relation. In our study, patients in the two clusters had the same GPs’ density. However, this result has to be interpreted with caution. The density of GP reflect healthcare availability, but not real healthcare access. No information related to the number of Nephrologists consultations/visits available per inhabitant per municipalities are available actually. Moreover, we found that means of transport differed significantly between clusters. This repartition urban and rural of the two clusters could explain differences in mods of transports used between clusters. Transportation costs are a very important part of the cost of dialysis in France and the reimbursement rates for medical transports depend on the urbanization of the municipality: urban or rural sector [5].

Factors related to social deprivation of the municipality also influenced the geographic distribution of patients starting dialysis in emergency. The socio-economic level of municipalities located in the higher-risk cluster was lower than in those within the lower-risk cluster. Previous works have documented an association between the level of deprivation of the residence’s area, based on income [34,35,36,37], education [34,35], composite socio-economic score [22,38], poverty [39,40], and unemployment [36], and the incidence of ESRD, RRT [34,38], and CKD [41,42,43]. The degree of deprivation of one neighborhood could be a potential determinant of access to healthcare. More deprived neighborhoods have more difficult access and fewer available of healthcare facilities [44]. However, in France, medical and hospital costs for patients with ESRD (the access to diagnosis and treatment of ESRD) are completely covered (100%), and the reimbursement is regulated by uniform rates regardless of whether the patient is treated in the public or private nephrology facility.

That is why previous French studies [33,45], hypothesize that beyond access to healthcare, the degree of deprivation of the neighborhood could be a potential determinant of factors that might influence the occurrence and progression of CKD like better preventive medical care from patients lived in lower deprived neighborhoods. No information is available to measure this hypothesis in our REIN registry, but previous studies highlighted that people with a relatively high level of education and who exercise a higher-level occupation are more likely to pay attention to their health and to warning signs of disease, particularly by monitoring blood pressure and nutritional changes. More educated individuals are also better equipped to find their way in the healthcare system, and to gain access to the best therapies [45]. They are also better informed on their disease.

### Limitations and Strengths

Several study limitations can be noted, starting with the study design. This is an ecological study, and therefore we must acknowledge the possibility of ecological fallacy bias. In fact, individual members of a municipality all have the average characteristics of the group as whole, when in fact any association observed between variables at the group level does not necessarily mean that the same association exists for an individual plucked from the municipality. Using municipality level data to identify areas at higher risk of emergency first dialysis certainly resulted in some loss of precision concerning the intra-municipality variability risk. The results obtained at the municipality level cannot be extrapolated to the individual level. Another limitation is the absence of the medical reasons underlying the nephrologist’s decisions to start dialysis in emergency because they are not recorded in the REIN registry. However, REIN registry included a quality control of their data. A clinical research assistant in each region visits every dialysis center to verify the completeness of patient and event registration, by comparing reports to the registry with center administration files.

This study also has several strengths, including the use of statistical tools in spatial epidemiology for the analysis of spatial information relevant to disease and of geographic and individual data concerning risk factors. This methodology allowed us to identify areas at higher/lower risk of emergency first dialysis in patients with ESRD after adjustment for sex and age, and provided a relevant map of the geographic distribution of the emergency first dialysis risk. This will be useful for improving healthcare policies in the region by targeting the potentially involved risk factors.

Moreover, data were extracted from the REIN registry that provides an exhaustive record of patients treated by RRT in France. This allowed us to include all patients who underwent emergency first dialysis, and all major comorbidities. Additionally, several categories of patient- and municipality-level factors potentially implicated in the risk of emergency first dialysis were included in the analysis.

Future comprehensive studies on the municipalities located in the higher-risk cluster could allow identifying modifiable causes of emergency dialysis start (e.g., healthcare organization, patient management, and observance) and could help setting up targeted educational programs.

## 5. Conclusions

This study showed geographic variations of emergency first dialysis risk in patients with ESRD living in Bretagne: one higher-risk municipality cluster in the western part of the region, and one lower-risk cluster in the eastern part. The patients’ clinical characteristics cannot explain this geographic variation. Conversely, the municipality where patients live seems to play an important role. Although the socioeconomic characteristics of the municipality where patients live are not sufficient to fully explain the variations in emergency first dialysis incidence, rurality, and longer time to reach the dialysis facility could partly explain these differences.

Spatial analysis is suitable for identifying high-risk areas and for disease surveillance. The results obtained with this method might allow health authorities to design relevant preventive actions and to develop programs to decrease the rate of emergency first dialysis.

## Figures and Tables

**Table 1:** Description of the clinical and biological characteristics of the patients with ESRD who started the first dialysis in emergency in the entire Bretagne region and within the higher-risk and lower-risk clusters (no. of patients = 665).

Variables	Entire Bretagne (*n* = 665)	Higher-Risk Cluster ** (*n* = 70)	Lower-Risk Cluster ** (*n* = 78)	*p*-Value *
Incidence of emergency first dialysis per 100,000 inhabitants (mean ± SD) **	270.3 ± 744.6	434.1 ± 573.2	133.1 ± 396.6	<0.0001
PRIMARY RENAL disease (%)				
Polycystic disease	3.8	0	1.3	0.980
Hypertensive and vascular nephropathy	26.4	12.9	25.6	0.051
Glomerulonephritis	12.3	7.1	19.2	0.052
Pyelonephritis	5.9	5.7	5.1	0.973
Diabetic nephropathy	9.6	8.6	14.1	0.316
Others (Unknown and unclassifiable nephropathy)	42	65.7	34.6	<0.0001
COMORBIDITIES (%)				
Respiratory disease	20.9	18.8	23.4	0.482
Active malignancy	22.2	33.3	20	0.09
Hepatic disease	4.6	5.9	5.2	0.940
Diabetes	31.5	28.6	32	0.722
CARDIOVASCULAR disease (%)				0.921
No	35.4	34.3	32	
One	21.2	22.9	21.8	
Two or more	43.4	42.9	46.2	
PHYSICAL DISABILITIES (%)				0.019
No	93.2	97.1	85.9	
One or more	6.8	2.9	14.1	
PSYCHIATRIC DISORDER (yes) (%)	26.8	33.3	26.3	0.901
BMI (%)				0.658
Underweight and normal (BMI < 25)	52.7	64.6	57.1	
Overweight (BMI ≥ 25)	29.8	23.1	28.6	
Obese (BMI > 30)	16.7	11.4	14.1	
SMOKING status (%)				0.849
Ex-smoker	46	44.7	50	
Smoker	17.6	14.9	13.9	
Never Smoked	36.4	40.4	36.1	
ALBUMIN (%)				0.444
<30 g/dL	41.1	35.7	26.5	
≥30 g/dL	58.9	64.3	73.5	
HEMOGLOBIN (%)				0.773
<10 g/dL	58.1	48.8	47.9	
10–12 g/dL	37.4	44.2	47.9	
>12 g/dL	4.4	7	4.1	

* *p*-values are calculated comparing proportion of patients in the high-risk cluster group to the low-risk cluster group of municipalities in each covariate using Chi-square test or exact Fisher test according to the assumptions. ** The higher- and lower-risk clusters were identified by SaTScan software as having significantly elevated or decreased risk of emergency first dialysis.

**Table 2:** Description of the socio-economic characteristics and ESRD management characteristics of the patients with ESRD who started the first dialysis in emergency in the entire Bretagne region and within the higher-risk and lower-risk clusters (no. of patients = 665).

Variables	Entire Bretagne (*n* = 665)	Higher-Risk Cluster ** (*n* = 70)	Lower-Risk Cluster ** (*n* = 78)	*p*-Value *
Incidence of emergency first dialysis per 100,000 inhabitants (mean ± SD) **	270.3 ± 744.6	434.1 ± 573.2	133.1 ± 396.6	<0.0001
SOCIO-DEMOGRAPHIC				
PROPORTION of MEN (%)	68.3	65.7	69.2	0.648
AGE by group (%)				0.579
<45 years	15.5	41.4	48.7	
45–75 years	49.6	40.0	32.1	
>75 years	34.9	18.6	19.2	
PATIENT’S ACTIVITY (%)				0.304
Inactive	85	85.5	78.9	
Active	15	14.5	21	
ESRD MANAGEMENT				
TIME in TRANSPORT (mean ± SD in minutes)	24.4 ± 0.7	21.5 ± 18.8	15 ± 11.1	0.0132
TYPE of TRANSPORT (%)				<0.0001
Ambulance	7.6	6.7	10.5	
Light sanitary vehicle	64.4	71.7	37.3	
Taxi	20.1	11.7	32.8	
Car	3.6	0	10.4	
Others	4.4	10	9	
VASCULAR ACCESS procedure (%)				0.939
Temporary vascular access	98	98.6	98.7	
Permanent vascular access	1.9	1.4	1.3	
N° of nephrologist consultations (%)				0.371
No consultations	46.4	58.3	46.4	
<3 consultations	17.6	11.7	13.0	
≥3 consultations	36.0	30.0	40.6	

* *p*-values are calculated comparing proportion of patients in the high-risk cluster group to the low-risk cluster group of municipalities in each covariate using Chi-square test or exact Fisher test according to the assumptions. *p*-values are calculated comparing means or ranks of the distribution between the two groups using *t*-test or Wilcoxon test according to the assumptions. ** The higher and lower-risk clusters were identified by SaTScan software as having significantly elevated or decreased risk of emergency first dialysis.

**Table 3:** Description of the socio-demographic characteristics of the municipalities in the entire Bretagne region and within the higher-risk and lower-risk clusters (no. of municipalities = 1270).

	Variables	Entire Bretagne (*n* = 1270)	Higher-Risk Cluster ** (*n* = 53)	Lower-Risk Cluster ** (*n* = 169)	*p*-Value *
	SOCIO-DEMOGRAPHIC				
Occupation	Proportion of blue-collar workers in the labor force (%)	34.1 ± 12.8	28.8 ± 8.9	28.9 ± 10.3	0.65
	Proportion of managers in the labor force (%)	8.6 ± 5.8	9.1 ± 5.5	13.4 ± 6.6	<0.001
	Proportion of intermediary workers in the labor force (%)	48.4 ± 11	50.7 ± 7.7	50.6 ± 7.8	0.11
Education	People aged 15 years or older with a higher education degree (%)	22.5 ± 6.8	23.3 ± 5.4	28.6 ± 8.0	<0.0001
	People aged 15 years or older with at least a lower tertiary education (%)	47.5 ± 4.8	48.3 ± 4.1	45.9 ± 4.9	0.002
	People aged 15 years or older who did not go beyond elementary education (%)	29.9 ± 7.2	28.3 ± 6.3	25.5 ± 6	0.006
Unemployment	Proportion of unemployed people (%)	10.2 ± 3.4	10.9 ± 2.8	7.8 ± 2.1	<0.001
Immigration status	Proportion of foreigners in the total population (%)	1.9 ± 2.5	1.2 ± 0.7	1.3 ± 1.1	0.49
Housing	Subsidized housing among all primary residences (%)	4.9 ± 4	6.8 ± 3.3	6.6 ± 4.7	0.37
	Household with at least one car (%)	91.9 ± 5.7	91.2 ± 7.4	93.7 ± 3.1	0.01
Resources	Median income per consumption unit (mean ±SD)	19,437 ± 2029	19,863 ± 1046	20,831 ± 2129	0.002
Residential mobility	Proportion of principal residence for less than 2 years (%)	10 ± 3.1	8.6 ± 2.8	11.3 ± 3.4	<0.001
	Proportion of principal residence for more than 10 years (%)	56. ± 6.7	60.7 ± 6.7	51.2 ± 7.1	<0.001

* *p*-values are calculated from the Chi-square test or exact Fisher test except for the median income were the Wilcoxon rank test was used comparing high-risk cluster group to low-risk cluster group of municipalities in each covariate. ** The higher and lower-risk clusters were identified by SaTScan software as having significantly elevated or decreased risk of emergency first dialysis.

**Table 4:** Description of the degree of urbanization and potential healthcare offer characteristics of the municipalities in the entire Bretagne region and within the higher-risk and lower-risk clusters (no. of municipalities = 1270).

Variables	Entire Bretagne (*n* = 1270)	Higher-Risk Cluster ** (*n* = 53)	Lower-Risk Cluster ** (*n* = 169)	*p*-Value *
Degree of URBANIZATION				
Urban (%)	17.2	24.5	23.1	**<0.0001**
Peri-urban (%)	5.3	0	10.6	
Rural (%)	77.6	75.5	66.3	
POTENTIAL HEALTHCARE OFFER				
Proportion of municipalities with at least one dialysis facility, number (%)	36 (2.8%)	3 (5.6%)	2 (1.2%)	**0.09**
General practitioners’ density per 10,000 inhabitants	5.5 ± 7.5	7.6 ± 9.3	5.5 ± 8.2	**0.09**

* *p*-values are calculated from the Chi-square test or exact Fisher test except for the GP density where the Wilcoxon rank test was used comparing high-risk cluster group to low-risk cluster group of municipalities in each covariate. ** The higher and lower-risk clusters were identified by SaTScan software as having significantly elevated or decreased risk of emergency first dialysis.

## Abbreviations

ESRD	End Stage Renal Disease
CKD	Chronic Kidney Disease
RRT	Renal Replacement Therapy
RR	Relative Risk
BMI	Body Mass Index
INSEE	National Institute of Statistics and Economic Studies
REIN	Réseau Epidémiologie et Information en Néphrologie
